# Crack nucleation using combined crystal plasticity modelling, high-resolution digital image correlation and high-resolution electron backscatter diffraction in a superalloy containing non-metallic inclusions under fatigue

**DOI:** 10.1098/rspa.2015.0792

**Published:** 2016-05

**Authors:** Tiantian Zhang, Jun Jiang, Ben Britton, Barbara Shollock, Fionn Dunne

**Affiliations:** 1 Department of Materials, Imperial College London, London SW7 2AZ, UK; 2 WMG, University of Warwick, Coventry CV4 7AL, UK

**Keywords:** crystal plasticity, nickel, decohesion, fatigue crack nucleation, interfacial strength

## Abstract

A crystal plasticity finite-element model, which explicitly and directly represents the complex microstructures of a non-metallic agglomerate inclusion within polycrystal nickel alloy, has been developed to study the mechanistic basis of fatigue crack nucleation. The methodology is to use the crystal plasticity model in conjunction with direct measurement at the microscale using high (angular) resolution-electron backscatter diffraction (HR-EBSD) and high (spatial) resolution-digital image correlation (HR-DIC) strain measurement techniques. Experimentally, this sample has been subjected to heat treatment leading to the establishment of residual (elastic) strains local to the agglomerate and subsequently loaded under conditions of low cyclic fatigue. The full thermal and mechanical loading history was reproduced within the model. HR-EBSD and HR-DIC elastic and total strain measurements demonstrate qualitative and quantitative agreement with crystal plasticity results. Crack nucleation by interfacial decohesion at the nickel matrix/agglomerate inclusion boundaries is observed experimentally, and systematic modelling studies enable the mechanistic basis of the nucleation to be established. A number of fatigue crack nucleation indicators are also assessed against the experimental results. Decohesion was found to be driven by interface tensile normal stress alone, and the interfacial strength was determined to be in the range of 1270–1480 MPa.

## 1. Introduction

Nickel-based superalloys are widely used for turbine disc applications. Modern superalloys with exceptional high temperature properties are produced via a powder metallurgy (PM) route in order to minimize micro/macrochemical segregation [1]. However, although great efforts have been made to reduce contamination introduced during manufacturing steps, non-metallic inclusions are inevitable in these alloys, resulting in degradation of mechanical properties and scatter in fatigue life. To address this issue, a series of studies have been carried out [2,3] to investigate the effects of inclusions under various conditions of loading, but it remains clear that crack nucleation occurring at agglomerate inclusions remains a significant technological and scientific challenge [4], and this paper addresses the mechanistic basis by which it occurs in the nickel superalloys.

The presence of non-metallic inclusions is common to a range of alloys in aluminium [5–7], steels [8] and nickel superalloys [9]. In the presence of these inclusions, cracks are rarely observed to nucleate in the surrounding metal matrix, but at the oxide inclusions either by oxide/metal decohesion or by fracture of oxide particles. Experimental observations indicate that such crack nucleation occurs very early and eventually leads to the development of cracks into the neighbouring microstructures.

Interface decohesion has been studied extensively using cohesive zone modelling. The cohesive zone theory relies on a phenomenological continuum framework with constitutive equations that relate surface traction and displacement. Complete separation occurs when the cohesive traction across the decohering interfaces becomes zero [10]. The predictive capability of such a damage initiation model relies on accurate prediction of stress and strain fields at appropriate length scales. However, in the literature, criteria for void nucleation at interfaces are related to and defined with respect to macroscopic quantities [11] and typically, in these analyses, the constitutive behaviour of each phase present is not considered explicitly. Cohesive zone models with interface potential functions predicted by first principles density functional theory (DFT) simulations have been used for analyses of the atomistic separation of *γ*-Ni(Al)/Al_2_O_3_ interfaces [12,13], and such strategies have been employed to address fracture of bimaterial interfaces at various length scales. However, the use of DFT simulations to determine theoretical interfacial strengths for metal–ceramic systems is problematic, particularly when the detailed metallurgy at the interface often remains elusive. An alternative strategy is to determine the interfacial strengths at the granular length scale. The determination of cohesive zone properties at this scale has also remained problematic, but a potential solution exists by bringing together the results of detailed high (spatial) resolution digital image correlation (HR-DIC) and high (angular) resolution electron backscatter diffraction (HR-EBSD) measurements, together with microstructurally faithful crystal plasticity representations in order to extract out interface properties.

Recent advances in microstructure-sensitive crystal plasticity modelling in conjunction with experimental validation have provided understanding of damage nucleation in dual phase (DP) steels [14], aluminium alloys [15] and tantalum oligocrystals [16]. In addition, by virtue of detailed microstructure-level comparison of model predictions with independent experimental measurement, significant confidence has been developed in crystal modelling capabilities to capture quantitatively microstructure-level strain, stress, stress state and dislocation density [17–19].

In previous studies [17,20], thermal residual strains and dislocation densities near non-metallic inclusions have been investigated by HR-EBSD and HR-DIC carried out in [20] have provided microscopic mapping of strain fields local to non-metallic inclusions. In the literature, full-field mapping of elastic strains and dislocation densities has been achieved by HR-EBSD in a wide variety of alloys [21–23]. DIC and EBSD characterization has recently been applied for studies of strain partitioning between ferrite and martensitic phases in steels [14], and strain heterogeneities and lattice rotations in 304L stainless steel [24]. The HR-DIC and HR-EBSD techniques used in the two consecutive analyses in [17,20] have enabled concurrent mapping of elastic strain, plastic strain and dislocation density at key microstructural features.

In this paper, we address the mechanistic basis of crack nucleation in nickel superalloy RR1000 containing oxide particle inclusions under fatigue. Concurrent crystal plasticity modelling is employed along with detailed HR-DIC (for total strain measurement), EBSD (for elastic strain and lattice rotation measurement) and micromechanical three-point beam testing with fully characterized microstructure in order to identify and quantify cracks and their nucleation sites. Grain-by-grain assessments of slip, plastic strain accumulation through ratcheting, maximum principal and hydrostatic stresses and stored energy are examined in the context of experimentally observed fatigue crack nucleation sites in order to explain the mechanistic basis. In §2, the overall methodology is summarized, detailing the experimental investigation, the multiscale modelling to establish appropriate submodel boundary conditions and the crystal plasticity submodel explicit representation of the experimental beam microstructure. Computational and experimental comparison of results is presented to provide justification for, and confidence in, the model. This is followed by a systematic analysis of the slip, strain accumulation, stress and stored energy occurring at sites of fatigue crack nucleation, and the assessment of the mechanistic basis for crack nucleation. In so doing, we present the extracted interfacial strength between the nickel matrix and oxide particles forming the agglomerate inclusions in RR1000.

## 2. Materials and methodology

### (a) Experimental methodology

#### (i) Thermal treatment

**Figure 1.**
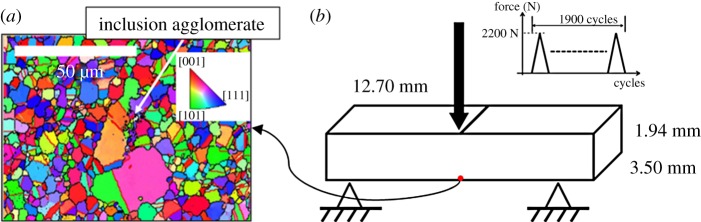
(*a*) EBSD map of inclusion agglomerate in polycrystalline nickel superalloy. (*b*) Schematic of the three-point bending beam. (Online version in colour.)

A RR1000 polycrystalline nickel-based superalloy sample was supplied by Rolls-Royce plc. The sample was produced via a PM route, followed by extrusion, forging and a two-step heat treatment. First, subsolvus heat treatment at 1393 K for 4 h was carried out followed by forced air quench. The sample was then aged at 1033 K for 16 h prior to slow cooling to room temperature. A non-metallic inclusion was detected in the nickel sample, which was then machined to leave the inclusion on the free surface. Metallographic grinding and polishing were carried out to obtain a surface finish required for secondary electron microscopy (SEM) and EBSD. This nickel/inclusion combination is shown in figure 1*a*. Upon cooling from ageing temperature to room temperature, the establishment of thermal residual strains and densities of geometrically necessary dislocations (GNDs) at inclusion/nickel matrix interfaces resulting from differing coefficients of thermal expansion was anticipated and analysed using HR-EBSD which is reported within a previous study [20].

#### (ii) Mechanical cyclic three-point bend test

The thermally treated nickel sample was subjected to three-point bend tests under force-controlled loading with a triangular *R*=0 wave form peaking at 2200 N as shown in figure 1*b*. The sample was removed from the loading rig for DIC measurement after selected intervals and then reloaded. Slip localization and elastic straining were studied using HR-DIC and HR-EBSD. Heterogeneous distributions of plastic strains during the fatigue loading were quantified using HR-DIC [20]. The measurements of thermal residual (elastic) strains, GND densities and cyclic plastic strains were related to observed fatigue crack nucleation in the tested sample. Decohesion of the oxide/matrix interface and particle fracture were the two crack nucleation mechanisms observed, and under the loading considered these were found to occur at a very early stage of the test (after two cycles).

In this study, the experimentally assessed sample briefly reviewed above is revisited and a geometrically faithful crystal plasticity finite-element model (FEM) developed for its direct and explicit microstructural representation. The microstructurally faithful model is subjected to an identical heat treatment and subsequent cyclic loading conditions in order to enable direct comparison between the model predications and experimental observations and measurements made, so enabling a mechanistic assessment of the defect nucleation process. The crystal plasticity modelling methodology is described next.

### (b) Crystal plasticity modelling

#### (i) Thermoelastoplastic crystal plasticity modelling

In the small strain crystal plasticity framework, the deformation gradient, **F**, is multiplicatively decomposed into thermal, **F**_*θ*_, elastic, **F**_e_ and plastic, **F**_p_, parts where the elastic contribution captures the lattice stretch and rigid body rotation and the latter plastic term governs dislocation movement on slip planes.

2.1 $$\text{F}={\text{F}}_{\theta }{\text{F}}_{e}{\text{F}}_{p}.$$

**F** can be calculated using a rate formulation, where the total rate of deformation, **D**, can be determined using equation (2.2) where the elastic rate of deformation, **D**_e_, can be obtained using Hooke’s law and the plastic rate of deformation, **D**_p_, is specified by a slip rule.

2.2 $$\text{D}={\text{D}}_{e}+{\text{D}}_{p}+{\text{D}}_{\theta }.$$

Isotropic thermal deformation is assumed in the formulation of flow kinematics such that the thermal rate of deformation, **D**_*θ*_, is expressed as

2.3 $${\text{D}}_{\theta }=\alpha \dot{T}\text{I}$$

where *α* is the coefficient of thermal expansion, $\dot{T}$ the rate of change of temperature and **I** is the identity matrix.

The plastic rate of deformation is related to the plastic velocity gradient, **L**_p_, using

2.4 $${\text{D}}_{p}=\text{sym}({\text{L}}_{p}),$$

and the plastic velocity gradient evolves as a result of dislocation glide on slip planes, the rate of which is governed by the slip rates of dislocations on all slip systems

2.5 $${\text{L}}_{p}=\overset{n}{\underset{i=1}{\sum }}{\dot{\gamma }}^{i}(\text{s}⊗\text{n}),$$

where ${\dot{\gamma }}^{i}$ is the rate of dislocation slip on the *i*th slip system with slip direction, **s** and slip plane normal, **n**. The physically based slip rule used within this study accounts for dislocation motion overcoming pinning obstacles by means of thermal activation and has been well reported [25]

2.6 $$\dot{\gamma }=\rho_{\mathrm{SSD}}^{m}b^{2}v\;\exp\left(-\frac{\Delta H}{kT}\right)\sinh\left(\frac{(\tau -\tau_{c})\gamma_{0}\Delta V}{kT}\right),$$

where *ρ*^m^_SSD_ is the density of gliding dislocations, *b* is the magnitude of Burger’s vector, *ν* is the frequency of jumps to overcome obstacle barriers, successful or otherwise, Δ*H* is the Helmholtz free energy, *k* is the Boltzmann constant, *T* is the temperature in kelvin, *γ*_0_ is the work conjugate to the resolved shear stress at the reference state and Δ*V* is the activation volume.

Isotropic hardening based on Taylor’s dislocation model [26] is assumed in the constitutive equation and shown in equation (2.7), where *G* is the shear modulus, *ρ*^s^_SSD_ is the density of sessile statistically stored dislocations and *τ*_0_ is the initial critical resolved shear stress. The hardening rate on each of the 12 fcc slip systems is assumed to be the same.

2.7 $$\tau_{c}=\tau_{0}+Gb\sqrt{\rho_{\mathrm{SSD}}^{s}}.$$

The density of the statistically stored dislocations is determined using a phenomenological relation with effective plastic strain based on experimental observation of the evolution of dislocation density. λ governs the rate at which the dislocation density evolves and thereby being material dependent and requiring calibration

2.8 $${\dot{\rho }}_{\mathrm{SSD}}^{s}=\lambda \dot{p},$$

where the rate of effective plastic strain is related to the plastic velocity gradient by

2.9 $$\dot{p}={\left(\frac{2}{3}{\text{L}}_{p}:{\text{L}}_{p}\right)}^{1/2}.$$

#### (ii) Identification of material parameters and physical properties

Uniaxial tensile tests on polycrystalline RR1000 samples without inclusions were carried out at Rolls-Royce plc. Identification of the slip rule critical resolved shear stress and isotropic hardening parameter, λ, was carried out using the tensile test data and a representative pseudo-three-dimensional finite-element (FE) model consisting of 100 crystals. The slip strength and hardening parameter in equations (2.6) and (2.8) were found such that the experimentally obtained stress–strain curve was well captured by the model. The full set of physical properties in the slip rule are listed in table 1. The Helmholtz free energy Δ*H* was chosen to generate undetectable rate sensitivity at the applied rate of loading (approx. 4.82×10^−3^ s^−1^) while being physically reasonable.

**Table 1.** Material parameters and physical properties for the nickel-based superalloy slip rule.

*ρ*^m^_SSD_	0.05 μm^−2^
*B*	3.51×10^−4^ μm
*V*	1.0×10^11^ s^−1^
Δ*H*	3.456×10^−20^ J
*K*	1.38×10^−23^ J K^−1^
*T*	293 K
*γ*_0_	8.33×10^−6^
*τ*_c_	450 MPa
**λ**	150 μm^−2^

#### (iii) Thermal modelling of heat treatment

**Figure 2.**
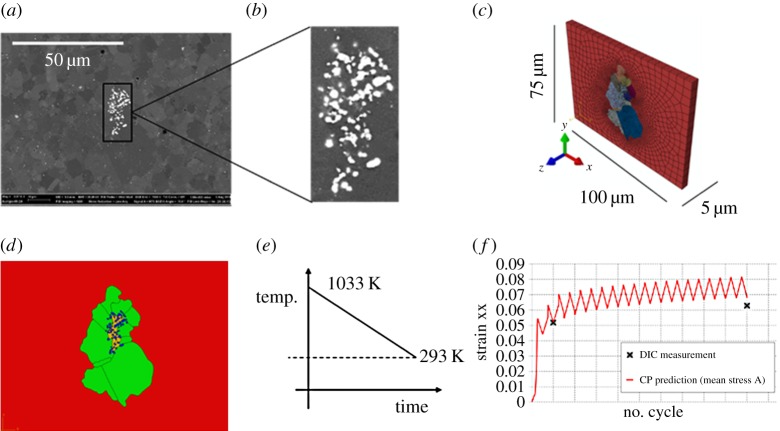
(*a*) BSE image of nickel polycrystal containing an inclusion agglomerate with (*b*) a magnified view showing the oxide particles which were modelled explicitly in (*c*) three-dimensional crystal plasticity finite-element model. (*d*) Front view of the crystal plasticity model with colours indicating the oxide particles, fine-grained nickel, coarse-grained nickel and surrounding elastic medium. (*e*) The applied thermal loading and (*f*) the calculated end experimentally measured (HR-DIC) cyclically evolving average xx strain obtained at the agglomerate region. (Online version in colour.)

The backscattered electron (BSE) image of the investigated region is shown in figure 2*a*, together with a magnified view of the inclusion agglomerate in figure 2*b*. A full and explicit geometrical FE representation of the inclusion/polycrystal matrix combination was developed using approximately 31 000 20-noded three-dimensional quadratic user-defined elements. The FE representation of the agglomerate-containing microstructure is shown in figure 2*c* in which the agglomerate, comprising a distribution of oxide particles within a fine-grained nickel matrix region may be seen, together with the coarse-grained nickel matrix surrounding it. The colours in figure 2*c* represent the differing measured crystallographic orientations implemented in the crystal model detailed below. In the model shown in figure 2*c*, the boundary conditions imposed are such that the left face is fixed in the *x*-direction, the bottom face fixed in the *y*-direction and the back face fixed in the *z*-direction. The boundary condition on the front free surface is therefore that of approximately plane stress in nature and reproduces the assumption made in the HR-EBSD measurement where the out-of-plane normal stress is reasonably taken to be zero.

A simplification made is that the grains are assumed to be prismatic through the depth of the FE model owing to the absence of information on subsurface grain geometry. This form of simplified representation has been used by other authors [15,27] to study microdeformation of polycrystals and reasonable agreement with experimental observations has been obtained. Other strategies for simplification of the microstructural representation include (i) using a substrate of randomly orientated grains underneath the surface grains of interest [28] and (ii) a layer of material with isotropic, homogeneous properties arranged below the free surface grains [29]. In the Ni alloy sample investigated in this work, the sizes of the surrounding abnormal grains are significantly larger than those in the fine-grained matrix, with the result that the subsurface fine grains beneath the abnormal grains likely play a small role in influencing the measured free-surface response. DIC measurements against model predictions to be shown later support this argument.

In figure 2*d*, the large grains surrounding the inclusion agglomerate (green) are modelled using elastically anisotropic crystal plasticity with their orientations specified with the knowledge of the crystallographic orientations obtained by the EBSD. The oxide particles (blue) are assumed to be elastic only. The small nickel grains local to the oxide particles (red) are modelled using elastically isotropic crystal plasticity. Largely owing to their number, but also size, their crystallographic orientations are uncertain and as a result, they are assigned a reference crystallographic orientation (i.e. [100] parallel to the global *x*-direction and [010] is parallel to the global *y*-direction). This region is anticipated to be dominated by plasticity, so while the full inclusion of the elastic anisotropy is desirable, it is argued to be of second-order importance. Outside of the coarse-grained region, the experimental microstructure shown in figure 1*a* shows the existence of another fine-grained largely untextured polycrystal nickel region. This region is modelled using elastically isotropic crystal plasticity because it is reasonable to assume the random texture gives rise to isotropic elastic properties. Because the development of plasticity is anticipated to be significant such that many slip systems will be activated, the stress and strain response of this fine-grained region can be homogenized and modelled using a reference crystallographic orientation. This outer region is also shown in red in figure 2*d*. The oxide particles and nickel crystals have, of course, very different thermal and mechanical properties, and these are captured within the crystal plasticity model.

In figure 2*e*, the applied thermal loading is shown from 1033 K to room temperature which was applied to the model to reproduce the experimental cooling process imposed on the nickel sample.

#### (iv) Cyclic mechanical beam modelling

The agglomerate FE model was then subjected to subsequent cyclic loading in order to reproduce the experimental conditions. The FE model shown in figure 2*c*,*d*, of course, a subregion (containing the agglomerate) of the full three-point beam sample shown in figure 1*b*. Hence, it is necessary to establish the appropriate FE submodel boundary conditions in order to replicate those developed in the experimental beam bending test.

Appropriate boundary conditions were determined using a macrolevel Mises plasticity FE model of the three-point bending beam together with the crystal plasticity submodel. Details relating to the development of the macrolevel model and submodel and the determination of the appropriate submodel boundary conditions are given in appendix A. As an indication of the suitability of this model, we show only the resulting average xx-strain profile obtained from the microstructurally faithful crystal plasticity submodel and the corresponding xx-strain obtained from HR-DIC measurements (at cycles 2 and 20 only). In the experiment and simulation, the xx-strains are obtained by averaging over a suitable region near the agglomerate (as detailed within appendix), and results are shown in figure 2*f*. One set of calculated results is shown for strain versus cycles which correspond to submodel applied mean stress A. The key result of this approach is that applied mean stress A replicates the experimentally obtained strain evolution with cycles. We note that the HR-DIC results (*ex situ*) are captured only at the beam unloaded state, so that the minimum strain peak in the calculation shown is to be compared with these measurements. More details of the choice of appropriate local mean stress loading are detailed in appendix A.

In §3, the results of the crystal plasticity modelling together with experimental HR-DIC and HR-EBSD measurements are presented for the preliminary thermal loading of the agglomerate sample, followed by the secondary cyclic mechanical loading. These results are then used in the assessment of the mechanistic basis of fatigue crack nucleation.

## 3. Results

### (a) Thermal residual strains

**Figure 3.**
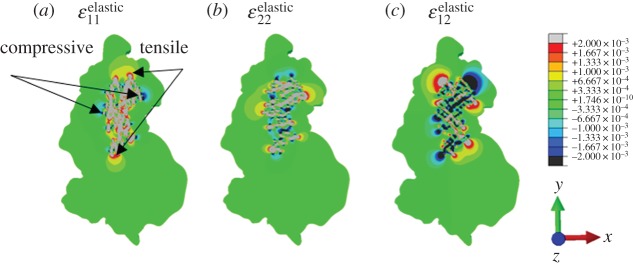
CPFE predicted elastic strain (*a*) xx, (*b*) yy and (*c*) xy components. (Online version in colour.)

During the final step of manufacturing the nickel sample, it was cooled from its annealing temperature of 1033 K to room temperature. Thermal residual strains develop at the inclusion/matrix interface owing to the difference in thermal expansivities. Thermal residual strains measured by HR-EBSD are purely elastic (owing to the nature of this technique). Therefore, dilatational strain and plastic strain have been removed from total strains in the CPFE predictions in order to make like-with-like comparisons. The CPFE model predicted elastic strains local to the agglomerate region are shown in figure 3*a*–*c*. Global field plots of the thermal strains demonstrate that compressive xx strains are observed near inclusion/matrix interfaces normal to the *x*-axis and tensile xx strains on interfaces parallel to the *x*-axis. The CPFE predicated yy strains are compressive near interfaces normal to the *y*-axis and tensile on interfaces parallel to the *y*-axis. In addition, expected shear strains with an antisymmetric distribution are shown in figure 3*c*.

**Figure 4.**
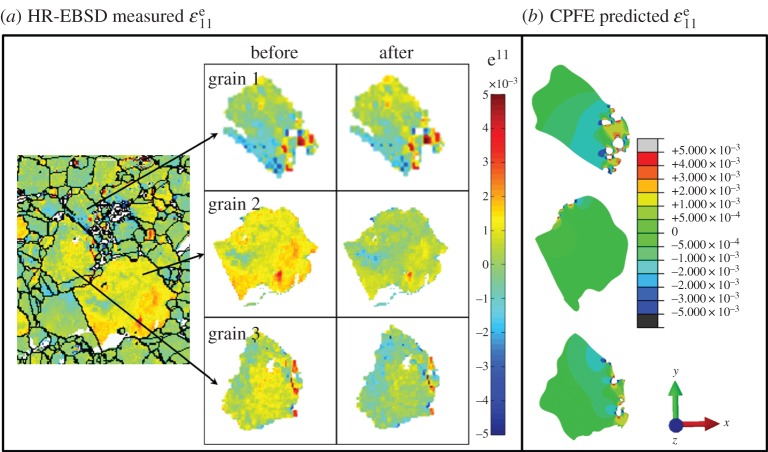
(*a*) HR-EBSD measured elastic strains before and after the direct reference shifting and (*b*) the corresponding elastic strains predicted by the CPFE model. (Online version in colour.)

A detailed comparison between the EBSD measurements and CPFE predictions is made by showing the elastic strains in three selected large grains near the inclusion agglomerate. In order to carry out these full-field comparisons, it is necessary to note that the EBSD elastic strains are measured with respect to an arbitrary reference point for each grain considered. Hence, direct reference shifting of the HR-EBSD strains to the corresponding point in the CPFE analysis has been carried out (the reference correction method [18]). The HR-EBSD measured elastic strains prior to (before) and after (after) direct reference shifting are displayed for completeness in the graphical table within figure 4*a*. Field plots of the elastic strains in the corresponding grains predicted by the CPFE model are displayed in figure 4*b*. The grain-average values of the CPFE predicted and HR-EBSD measured elastic strains in these three grains are given in table 2.

**Table 2.** Grain-averaged thermal residual strains in three selected grains in figure 4.

	CPFE predicted $\varepsilon_{xx}^{e}$	HR-EBSD measured $\varepsilon_{xx}^{e}$
grain 1	−2.53×10^−4^	−1.71×10^−4^
grain 2	−2.48×10^−4^	−5.87×10^−5^
grain 3	−5.97×10^−5^	−9.51×10^−5^

Qualitatively, there is good agreement between the directly shifted EBSD measurements and CPFE predictions. These elastic strains (and correspondingly, stresses) constitute the initial residual stress state at the agglomerate inclusion for the beam sample prior to subsequent cyclic loading and are therefore potentially important in the fatigue crack nucleation process.

**Figure 5.**
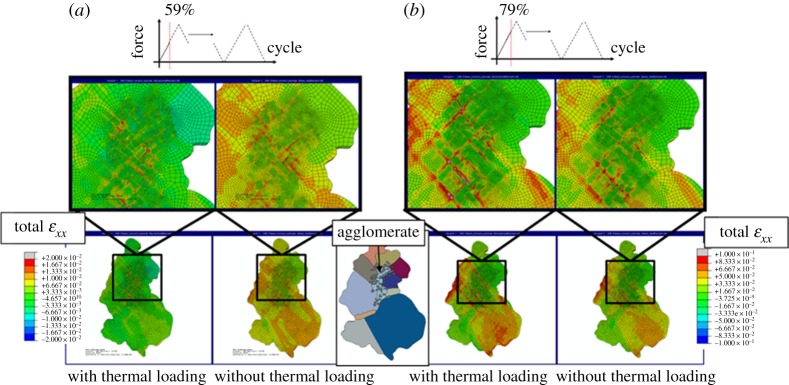
Field plots of total xx strain predicted by CPFE models with and without prior thermal loading at (*a*) *F*=0.59 *F*_max_ and (*b*) *F*=0.79 *F*_max_ in mechanical loading. The model aggomerate region microstructure is provided as an inset. (Online version in colour.)

The effects of thermal residual strains and dislocation density on subsequent fatigue loading are evaluated next. Microstructure-representative models both with and without thermal loading history have been subjected to subsequent representative stress-controlled mechanical loading. Field plots of total xx strain at two different stages (*F*=0.59 *F*_*max*_ and 0.79 *F*_*max*_) in the loading are shown in figure 5. Loading histories are given as insets.

Incorporation of thermal loading clearly has significant effects on the distribution of strains at an early stage in the fatigue test. In figure 5*a*, at *F*=0.59 *F*_max_, the distributions of xx strains in models with and without thermal loading are significantly different where strains are localized near the agglomerate in the former. Strain banding orientated at approximately 45° to the axial axis is formed within the agglomerate region in the model without prior thermal loading. The formation of such bands is due to constraints imposed by the agglomerate oxide particles included in the model, and shown in figure 2*b* within the experimental sample microstructure, which are assumed to deform elastically, and act as nucleators of localized slip and therefore of slip bands. However, the thermal residual stresses are found to play a diminished role at the later stage of the cyclic loading, as at *F*=0.79 *F*_max_ within the first loading cycle, the effects of the thermal residual strains have been largely minimized by the subsequent applied cyclic loading, evident from figure 5*b*.

### (b) Low cycle fatigue modelling and experimental observation of crack nucleation

**Figure 6.**
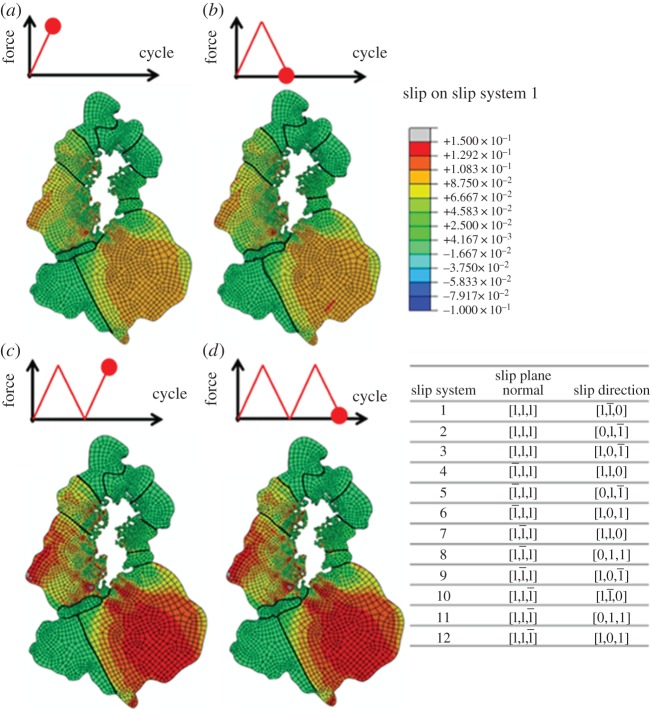
Field plots of slip on slip system 1 at times shown during the first two fatigue cycles. The inclusion agglomerate is shown in white. (Online version in colour.)

Accumulated slip on individual slip systems is potentially important both to indicate sites of crack nucleation and the cycles required. It is useful to examine changes in distributions of slip on each individual slip system in the course of the subsequent fatigue loading. Figure 6 shows the temporal evolution of slip, *γ*, for a dominant slip system (1) of the type (1 1 1) [1 $\bar{1}$ 0] with the other 11 slip systems for the fcc crystal numbered in the table included in the figure. The distributions of slip on slip system 1 are quite heterogeneous, and the level of heterogeneity increases progressively as a function of fatigue cycles. This observation is also seen for the other 11 slip systems, indicating that the heterogeneous strain distribution is established early during the low cycle fatigue, and no additional slip systems were subsequently activated. In this model, stress redistribution owing to plasticity with elastic anisotropy and geometrical constraints has not been observed to cause onset of secondary slip. Because the slip on each individual slip system contributes directly to the effective plastic strain, the localization of effective plastic strain is expected to become more pronounced, but the heterogeneous distribution remains persistent after even with further cyclic loading.

**Figure 7.**
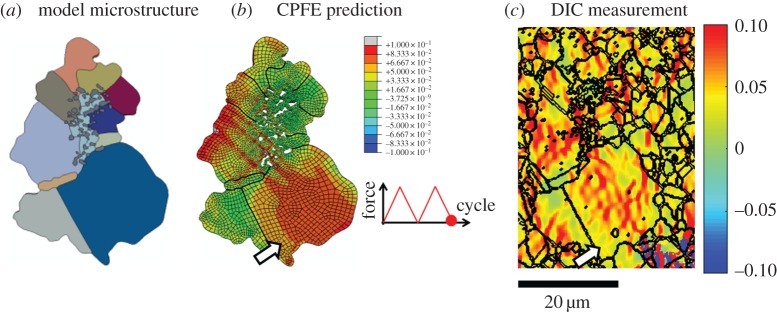
(*a*) Agglomerate inclusion and surrounding microstructure, (*b*) field plot of total xx strains after two cycles obtained by CPFE modelling and (*c*) total xx strains from DIC in the agglomerate region. (Online version in colour.)

The total xx strains after two cycles predicted by the CPFE model and those measured by HR-DIC are shown in figure 7*b* and *c*, respectively. Significant strain localization and heterogeneity is observed in the nickel grains in both the CPFE predictions and HR-DIC measurements, where the large grain identified in figure 7 by the arrow exhibits high strain levels in its interior in contrast to the regions close to the inclined grain boundary (GB) with low strain levels. Moreover, the strains measured by the HR-DIC indicate some localization of slip.

**Figure 8.**
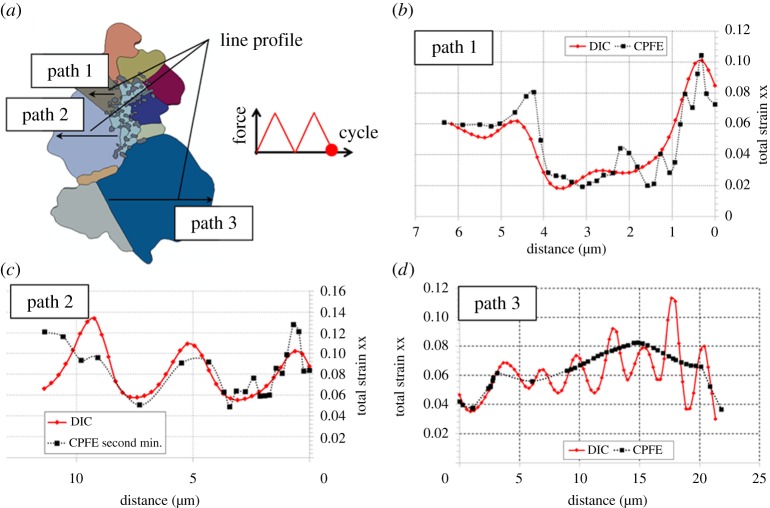
(*a*) The agglomerate inclusion model and the corresponding paths 1, 2 and 3 along which xx strains are obtained by HR-DIC and from the CPFE simulation extracted for (*b*) path 1, (*c*) path 2 and (*d*) path 3 at the end of the second cycle shown figure inset in (*a*). (Online version in colour.)

In order to facilitate quantitative comparison, xx strains from the CPFE model and experimental measurement have been extracted along three paths shown in figure 8*a* and plotted in figure 8*b*–*d*. Localized strain bands are observed in both numerical and experimental results for paths 1 and 2. However, along path 3 in a larger grain, the CPFE predicted pattern of strain localization is more uniform than that for the DIC measurements. The sharp gradients indicated by the DIC results may correspond to the formation of slip lines on the free surface which are therefore not captured by the CPFE model which is not able to capture the discrete nature of dislocation slip. However, in general, the detailed comparison of total strains developing from complex thermomechanical cyclic loading between model and experimental results at the local, microstructural level shows good agreement both qualitatively and quantitatively. It is argued that the model provides a sufficiently accurate account of empirically obtained strain measurement to make it suitable for further systematic study of the agglomerate inclusion, particularly in the context of understanding experimentally observed fatigue crack nucleation.

**Figure 9.**
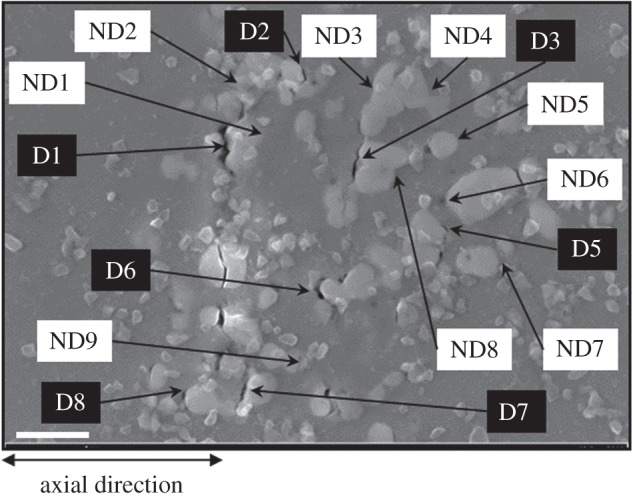
BSE image of the agglomerate inclusion after 1000 cycles. Locations where oxide/nickel matrix interface decohesion occurred are identified and numbered by black markers. Locations indicated by white markers are at oxide/nickel matrix interfaces, but are examples of interfaces where decohesion does not occur. Scale bar is 2 μm.

In a previous study [20], fatigue crack nucleation was experimentally observed to occur in the agglomerate sample considered in this paper via two mechanisms, namely decohesion of oxide/nickel interfaces and particle fracture. The full-field crystal plasticity modelling with qualitative and quantitative validation against experimental measurements is therefore used to provide a systematic study of the fatigue crack nucleation mechanisms in this sample. First, locations where interface decohesion were experimentally observed are identified and labelled in figure 9.

It is notable that the primary defect nucleation mechanism is that of oxide/metal decohesion, but some examples of particle cracking are also observed. These observations suggest that the process of nucleation is likely dominated by the behaviour of the oxide particles for which a stress-based failure mechanism is indicated because of their brittle nature. However, if this is so, then it is important to consider the role of the local adjacent nickel matrix slip accumulation, because previous studies [20] and this work have shown that strong local ratcheting occurs, generating significant hardening and hence higher stresses across the oxide/metal interfaces. In addition, in our mechanistic study, it is important to keep open the possibility that the local ratcheting also leads to the progressive development of dislocation accumulation, and consequently stored energy [30], potentially driving defect nucleation within the nickel matrix but at the metal/oxide interface. Hence, in what follows, these mechanisms are all assessed, but without favour towards any one initially, but the results obtained are then used to draw conclusions.

The parameters which are typically considered important for decohesion and cracking were calculated and assessed within the CPFE model at differing stages in the cyclic loading history, including the maximum principal stress *σ*_*I*_, the hydrostatic stress *σ*_H_, the effective plastic strain *p*, the incremental effective plastic strain Δ*p* (given by Δ*p*=*p*_*N*+1_−*p*_*N*_), total stored energy *G* and stored energy per cycle $\dot{\text{G}}$ (defined in [30]). These quantities, calculated by the CPFE model, at differing stages in the cyclic loading history, are assessed in association with the experimentally observed crack nucleation.

**Figure 10.**
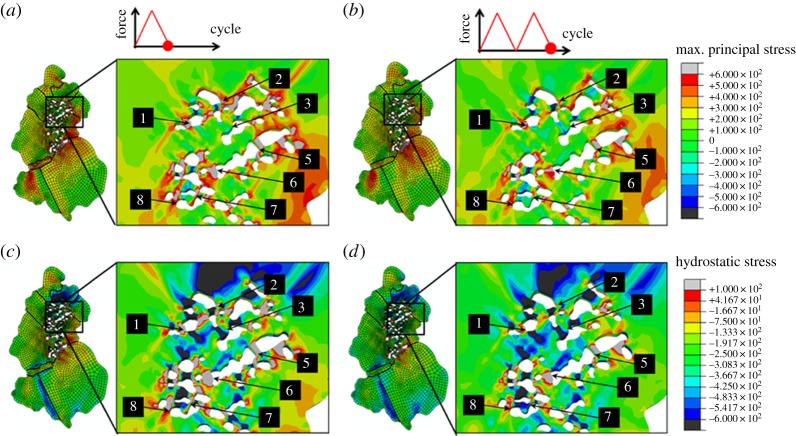
CPFE field plots of hydrostatic stress and maximum principal stress at the unloaded states of (*a*,*c*) the first cycle and (*b*,*d*) the second cycle with magnified views of the agglomerate regions in which experimentally observed oxide/matrix interface decohesion sites are labelled. (Online version in colour.)

In figure 10*a*–*d*, field plots of maximum principal stress and hydrostatic stress are shown for the unloaded states of the first and second cycles. The oxide particles are shown in white, and GBs are overlaid where appropriate and magnified views of the agglomerate region are shown in the insets. It is noted from the global field plots that crystallographic orientation together with GB constraint play an important role in the stress distributions. Discontinuous stress component distributions can be observed near GBs owing to the differing crystallographic orientations.

In the agglomerate region, the local distributions of the stresses are strongly affected by the geometries of the agglomerate particles where oxide/nickel interfaces perpendicular to the axial loading direction are likely to generate high tensile stresses, whereas interfaces parallel to the axial loading direction are likely to produce compressive stress states. It is noted that the complex local stress state near an interface, and the sometimes complex interface orientations, can generate maximum principal stress with a direction parallel to the tangent to the interface. For decohesion, an interface across which compressive normal stress has been established is considered to be infinitely strong, and therefore prohibit interface separation [31], and vice versa. For this reason, decohesion is observed to occur mostly at interfaces perpendicular to the axial loading direction.

Smaller interparticle spacing is found to cause an increase in the magnitude of stress. Considering the SEM image in figure 9, interface decohesion occurs at locations with high stresses, but they do not unambiguously match the locations with the highest predicted stresses. The effects of the second cycle on maximum principal and hydrostatic stresses may be assessed by comparing figure 10*b*,*d* with figure 10*a*,*c*, in which there are some visible changes in the distributions of the stresses between the first and second cycle. However, the magnitudes of stress very local to agglomerate particles can vary significantly (approx. 500 MPa). The stress localization in the agglomerate region after the second cycle appears to be less pronounced than that after the first cycle owing to the decrease in mean value of macroscopic stress. The above observations suggest stress is likely to be a necessary condition to cause interface decohesion.

**Figure 11.**
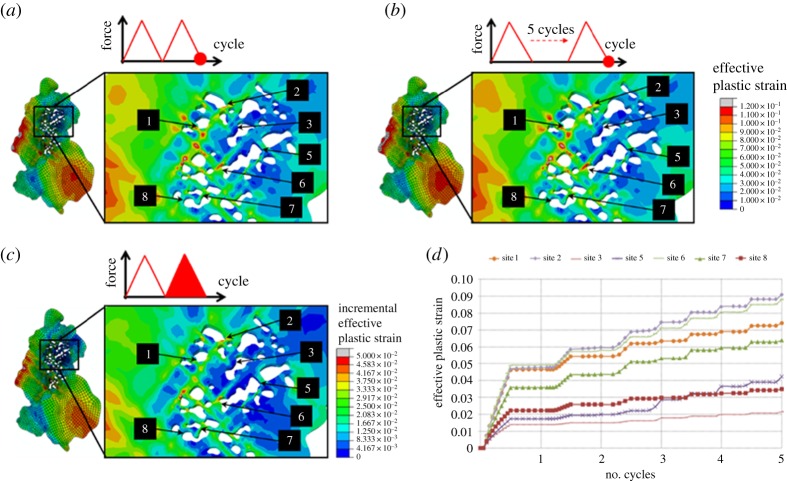
Field plots of accumulated plastic strain for the agglomerate region predicted by the CPFE model (*a*) after the second cycle and (*b*) after the fifth cycle and (*c*) incremental effective plastic strain developed within the second cycle. Evolution of effective plastic strain at locations where decohesion occurs is plotted in (*d*). (Online version in colour.)

Next, the accumulated plastic strain, *p*, calculated by integrating equation (2.9) is assessed for the unloaded states after the second and fifth cycles as shown in figure 11*a* and *b*, respectively. Following this, the incremental effective plastic strain, Δ*p*_*N*=2_, is evaluated and plotted in figure 11*c* for possible correlation with crack nucleation.

Magnified views of the agglomerate region are provided for point-by-point comparison with the debonded locations labelled in figure 9. The distributions of microplasticity in the coarse-grained agglomerate region are influenced strongly by the crystallographic orientation with respect to loading axis. The nickel grains to the left of the agglomerate are favourably orientated for slip and therefore exhibit higher effective plastic strains as seen in the magnified views in figure 11*a*,*b*.

The evolutions of effective plastic strains at locations where decohesion occurs are plotted in figure 11*d*. Although the rate of increase of plasticity in the region of interest decreases significantly after the second cycle as seen in figure 2*f*, plastic ratcheting is still observed locally at the agglomerate region, emphasizing the role of microstructural heterogeneities on development of localized microplasticity. The rates of accumulation of *p* are seen to be different from site to site. The effective plastic strain at site 8 is larger than at site 5 after the first cycle, but the rate of accumulation of *p* is larger at site 5 than at site 8. This results in greater plasticity at site 5 than at site 8 after five cycles. In sites 1, 3, 7 and 8, stabilization of *p* seems to have been achieved after just a few cycles. In relation to crack nucleation, a range of *p* and incremental Δ*p* values are found to coincide with the experimentally observed decohesion sites, and therefore neither *p* nor Δ*p* appear directly appropriate as indicators for the prediction of location of interface decohesion in this nickel sample (though it is recognized that the mechanistic basis associated with oxide/nickel interface decohesion is likely to be different to that for fatigue crack nucleation).

A stored energy quantity associated with the establishment of dislocation structures as a result of slip proposed in [30] for the basis for fatigue crack nucleation is evaluated next. Dislocation pile-up and slip localization are important in fatigue crack nucleation. For example, an energy approach based on the interaction between persistent slip bands and GBs for fatigue crack nucleation has been established in [32,33]. The formation of slip accumulation and dislocation structures gives rise to local stored energy, which has also been used as a failure criterion. The stored energy per cycle $\dot{\text{G}}$ is given by

**Figure 12.**
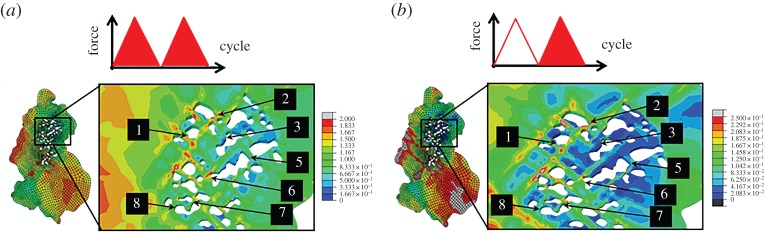
Field plots of the (*a*) energy stored in the first two cycles (*b*) stored energy rate for the second cycle. (Online version in colour.)

2.10 $$\dot{G}=\oint_{\mathrm{cycle}}\frac{\xi \sigma :d\varepsilon^{p}}{\sqrt{\rho_{\mathrm{SSD}}}},$$

where ***σ*** and ***ε***^p^ are the stress and plastic strain tensors, respectively, *ρ*_SSD_ is the density of statistically stored dislocations in equation (2.8), and *ξ* that fraction of the plastic dissipative energy which is stored (e.g. in the formation of dislocation structures), which is taken to be 0.05 based on the argument in [30]. The integration is carried out over a complete saturated fatigue cycle. In this study, the stored energy rate for the second cycle ${\dot{\text{G}}}_{N=2}$ and the energy stored in the first two cycles *G*_*N*=2_ are assessed and are shown in figure 12. Interface decohesion is found not to coincide with either local peak value of *G*_*N*=2_ nor ${\dot{\text{G}}}_{N=2}$, with the exception of site 2. Regions and locations with higher values of *G*_*N*=2_ and ${\dot{\text{G}}}_{N=2}$ can be found in the large surrounding nickel grains.

The field plots of hydrostatic stress, *p*, Δ*p*, *G* and $\dot{G}$ (figures 10–12) indicate that interface decohesion is unlikely to be controlled by any one of these quantities, because its occurrence is not predicted unambiguously at peak values of any one of these parameters. A condition for crack nucleation through interface decohesion has been discussed in [10,34], in which it is suggested interfacial strength and separation energy should be accounted for concurrently. Here, we assess the mechanistic basis for interface decohesion is through assessment of relationships between the model predicted *σ*_H_, *p* and *G*.

**Figure 13.**
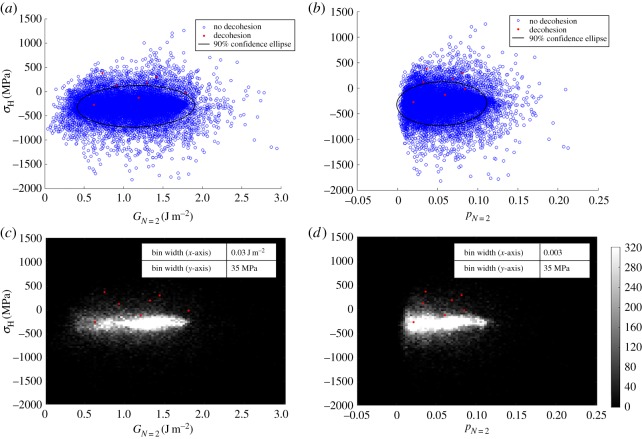
Hydrostatic stress as a function of (*a*) effective plastic strain and (*b*) stored energy with 90% confidence ellipse overlaid. The blue pointscorrespond to all nickel Gauss point data, and the red points are those within the nickel but at interfaces which show decohesion. Intensity plots of hydrostatic stress with respect to (*c*) stored energy and (*d*) effective plastic strain with data at sites of decohesion overlaid (red). All data correspond to unloaded state of second cycle. (Online version in colour.)

This is performed in figure 13 where the blue values correspond to all nickel Gauss point data in the FEM, whether at locations adjacent to an oxide interface or otherwise, and points in red are those within the nickel matrix show decohesion in the experiments. All data correspond to the unloaded state at the end of the second cycle. The ellipses shown within this figures indicate the 90% inclusion limit on all nickel points, and related to the ellipses.

The covariance of hydrostatic stress and effective plastic strain, as well as stored energy can be visualized as the angle between the *x*-axis and the ellipse major axis (figure 13*a*,*b*). In each case, the covariance is zero, as the hydrostatic stress is found to be correlated with neither effective plastic strain nor stored energy. The figures indicate that high hydrostatic stress is likely at sites for which oxide/nickel decohesion occurs, but this is not unambiguously necessary. They also show that at the sites of decohesion, the higher stresses occurring do so largely in the absence of, or independently from, high levels of either the effective plastic strain or the stored energy after two loading cycles (with the possible exception of one decohesion site and the latter).

Further statistical analysis on the correlation between hydrostatic stress and stored energy, as well as effective plastic strain are performed where the data are binned in hydrostatic stress (*x*-axis), stored energy (*y*-axis in figure 13*c*) and effective plastic strain (*y*-axis in figure 13*d*). The intensity corresponds to the count in each bin. In both intensity plots, the distributions further support that hydrostatic stress is correlated with neither stored energy nor effective plastic strain. In addition, it becomes clearer still that the stresses corresponding to the sites of decohesion are really quite well differentiated from the vast majority of non-decohering sites.

**Figure 14.**
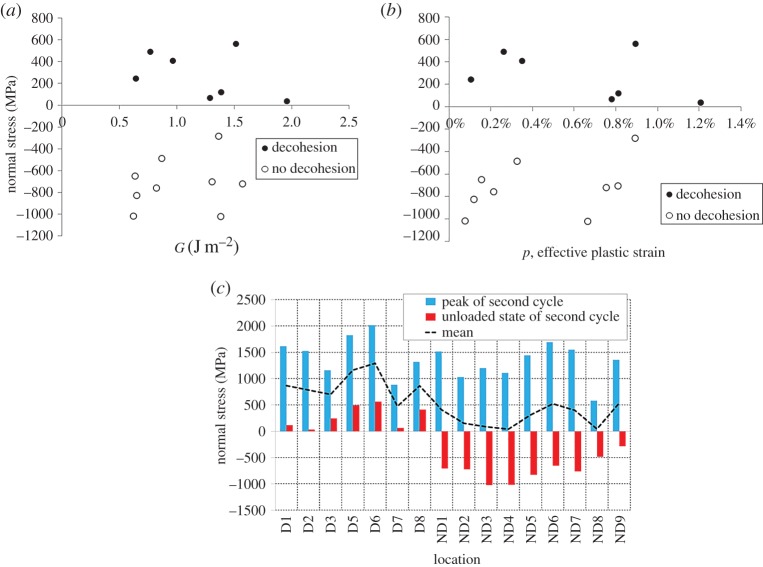
Normal stresses extracted at the locations labelled in figure 9 at the unloaded state of the second cycle plotted againstthe corresponding (*a*) stored energies and (*b*) effective plastic strains. (*c*) Normal stresses from peak (blue columns) and unloaded (red columns) states of the end of second cycle at the locations in figure 9. (Online version in colour.)

As a consequence of a lack of strong correlation of hydrostatic stress, effective plastic strain and stored energy, we return in more detail to the component of stress acting at oxide/nickel interfaces. We examine the effect of the stress normal to oxide/matrix interfaces on decohesion and examination of figure 9 shows that void formation occurred only at interfaces perpendicular to the axial direction. Normal stresses at the decohesion sites in figure 9 were extracted, at unloaded states at the end of the second cycle, and are plotted against the corresponding stored energy and effective plastic strain shown in figure 14*a*,*b* for interfaces that are normal to the axial loading direction. Not all perpendicular interfaces gave rise to decohesion. Figure 14*a*,*b* reveals that all decohesion sites exhibit tensile normal stress in both the fully loaded and unloaded states, which clearly differentiates them from those locations at perpendicular interfaces where decohesion does not occur. Both the decohered (tensile normal stress) and non-decohered (compressive normal stress) locations show broad ranges of stored energies and effective plastic strains, providing a strong indication that tensile normal stress is the principal requirement for decohesion.

In figure 14*c*, normal stresses from the peak and unloaded states of the second cycle are plotted for the marked locations in figure 9, which are sites on interfaces that are perpendicular to primary loading. We separate locations where decohesion occurs (D) and does not occur (ND). Average normal stresses were calculated and are shown as the broken line. Normal stresses from the peak of the second cycle (blue columns) are all tensile. At the unloaded state of the second cycle (red columns), only those normal stresses where decohesion occurs are tensile, whereas those at locations without cracking (ND1–ND9) are compressive. The average normal stress over peak and unloaded state at decohered locations is of magnitude approximately 500 MPa higher in tension than that for which decohesion does not occur. This higher average normal tensile stress drives the process of interface decohesion and can be used to estimate interface properties, which is addressed next.

Interface strength can be estimated by considering the average normal stress associated with the interfaces which show decohesion (D1–D8) in figure 14*c* and the average stress for those which do not (ND1–ND9) under the conditions of peak loading. Hence, the former gives the decohesion strength as 1480 MPa and the latter, the lower bound on interface strength as 1270 MPa. Hence, we conclude that the nickel/oxide interfaces have decohesion strength within this range.

## 4. Discussion

The objective in this work is to use fully integrated micromechanical test with experiments based upon HR-EBSD and HR-DIC characterization and simulation with microstructurally faithful crystal plasticity modelling. This enables realization of new insights into the mechanistic basis of crack nucleation in the context of polycrystal nickel alloy containing agglomerate inclusions.

Experimental observations make clear that the primary and dominant mechanism of crack nucleation is interfacial decohesion between nickel matrix and oxide particles, and that it occurs almost exclusively at interfaces which are arranged to be near normal to the primary remote stress. Fatigue crack nucleation in this material system was not observed to occur within the bulk of the nickel, neither in the fine-grained nor coarse-grained regions associated with the agglomerate inclusion. From this alone, the unambiguous conclusion is that the crack nucleation process is dominated simply by metal/oxide interfacial normal strength.

Combining these experimental observations together with simulations enables richer probing of microstructure and this study addresses the role of local slip accumulation, the development of slip localization or banding and local stored energy. This study reveals that the crack nucleation process in this material system remains independent of these quantities individually, often relevant in other fatigue studies [4,30]. The integrated experimental-CPFE modelling approach makes clear that the dominant mechanistic basis for crack nucleation is metal/oxide decohesion driven by the tensile stress normal to the interface. However, the role of local slip accumulation must also be recognized. Experimental evidence [30] and the CPFE results in this work demonstrate that slip accumulation by ratcheting local to the oxide particles occurs, leading to hardening and increasing stresses across the metal/oxide interface. Hence, in early cycles, the stresses may not be high enough to lead to decohesion, but with subsequent cycling and ratcheting, they progressively increase to exceed the fracture threshold and hence lead to decohesion. The integrated experimental-modelling approach has enabled the interfacial strength of the nickel/oxide particle boundaries to be estimated to be 1480 MPa with a lower bound of 1270 MPa. With the knowledge that stress-driven decohesion controls defect nucleation, and with the interface strength quantified, it becomes possible to establish safe maximum stress loading in service.

The calibration of the slip rule parameters was achieved by using experimental and modelled uniaxial tensile data. It has been demonstrated that evolution of dislocation densities and dislocation structures differs between cyclically deformed materials and their counterparts under monotonic tension [35,36]. Dislocation densities in cyclically deformed materials were observed to increase rapidly as a consequence of imposed strain and then saturate with fatigue cycles owing to interactions (annihilation of dislocations with opposite signs, cross-slip) [35,37,38], and the evolution of dislocation density was found to be associated with the formation of dislocation structures. In this study, cracks formed early under the applied cyclic loading and this is argued to occur by virtue of the increasing local dislocation densities at metal/oxide interfaces with slip ratcheting, pushing up local metal/oxide interface stresses.

The geometrical FEM established was developed by direct use of the EBSD orientation map and BSE image. Because these modes of imaging within the SEM are a surface characterization technique, microstructures in the out-of-plane direction are not easily accessible in a non-destructive manner and therefore they are assumed to be columnar during this model creation. This assumption is appropriate for the large surrounding grains, because a post-mortem cross sectioning of a similar agglomerate has indicated that the coarse nickel grains extend deeply through the depth [17]. Moreover, the microstructures underneath the agglomerate particles are not known, resulting in difficulties in explicit characterization and modelling. Therefore, the potential effects of such hard agglomerate particles, should they exist embedded underneath the surface, cannot be completely eliminated. Nevertheless, there is little doubt that the simplifying assumptions made potentially contribute to the differences obtained between the CPFE modelling predictions and experimental DIC measurements, and alternative microstructural modelling approaches [28,29] were discussed earlier. Methods for determination of three-dimensional microstructures, such as focused ion beam-EBSD [39,40] and synchrotron-based near field high energy X-ray diffraction microscopy [41,42], and differential aperture X-ray microscopy [43,44], are helpful to replicate through-depth grain morphologies.

Incorporation of length-scale effects by introduction of GND density can have significant influence on local plastic strain distribution. Previously, HR-EBSD and CPFE modelling studies have demonstrated the establishment of GND densities at carbide/metallic matrix interfaces during thermal cooling in nickel superalloys [18,19]. However, although the distributions of the predicted plastic strains were strongly influenced by incorporation of length-scale effects, the elastic strain distributions (and hence residual stresses) did not vary significantly from the HR-EBSD measurements. Because interface decohesion has been related to local stress state, it is argued that length-scale effects may not contribute significantly to this decohesion mechanism. Certainly, its influence on local strain/stress response and consequently stored energy would ideally need to be included in future studies.

In this study, interface decohesion has been demonstrated to correlate strongly with hydrostatic stress at the microstructural level. Prior work has indicated that void nucleation in commercially pure aluminium and Al2124 has shown that peak strains, i.e. critical strain, did not correlate well with particle/interface decohesion, using *in situ* tensile testing coupled with X-ray tomography in parallel with cohesive zone FEM [7]. Critical normal and hydrostatic stresses in a range of 220–1060 MPa were implemented in their FE analysis. Sensible strengths for ceramic/Al interfaces ranging from 200 to 750 MPa were suggested in [45] in connection with tensile deformation in a model composite.

In our study, decohesion was always found to occur at interfaces normal to the macroscopic loading axis, and a direct correlation with interface normal stress has been established in figure 14. Tensile normal stress local to and across the oxide/nickel interface has been found to be essential for decohesion.

Interface decohesion is the initial and likely primary fatigue crack nucleation mechanism in polycrystalline nickel superalloy containing agglomerates, as the oxide/nickel interface is brittle in comparison with other microstructural features, such as twin boundaries or hard particles. Therefore, the controlling micromechanical quantity is expected to differ from those for particle cracking and fatigue crack nucleation from other crystallographic features. The CPFE modelling has provided the contributions of accumulated slip and stored energy at the interfaces (and elsewhere), as shown in figure 13. Stored energy is associated with the accumulation of plastic slip together with stress through hardening resulting from the establishment of dislocation accumulation. This occurs through accumulation of dislocations near the agglomerate oxide/nickel matrix interface.

Previous atomistic studies on traction-displacement for separation of a Ni/Al_2_O_3_ interfaces have been carried out by means of DFT in [13], and upscaling of the tractions obtained and the work of separation was used to implement and inform a macroscopic cohesive zone model. The atomistic traction/displacement relationship was used as the separation criterion. This required a critical normal or shear traction to be defined with the interatomic potential function playing the dominant role in determining interface separation.

The study presented supports the argument that the mechanistic basis of oxide/nickel interface decohesion may be well represented by use of cohesive zone models [10,46], which take into account interfacial strength and toughness. Some recent example studies use this approach well in r metal matrix composites [45] and in DP steels [47].

Complex chemistry local to agglomerate particle/nickel interfaces and coherency of the oxide/matrix interface at smaller length scales can have significant influence on the fracture behaviour of an interface. For example, the addition of Hf dopant on Ni/Al_2_O_3_ interfaces was assessed using first principles DFT [48,49] and a solid-state de-wetting experiment [50]. Beneficial effects of Hf segregation to the Ni/Al_2_O_3_ interfaces have been reported in [48,49] where the work of separation has been found to increase by a factor of three. However, Hf segregation did not occur in [50] owing to limited solubility and local oxygen partial pressure, which are in favour of formation of HfO_2_. In this context, depending on thermodynamic driving forces, microsegregation of differing alloying elements to the agglomerate particle/nickel interface can occur during heat treatment [51], leading to strengthening or weakening of the interface. There remains merit, therefore, in the indirect measurement approach presented, at least in the context of mechanistic understanding and in the establishment of engineering properties.

## 5. Conclusion

Crack nucleation through interface decohesion in a PM polycrystalline nickel superalloy containing a non-metallic agglomerate inclusion has been studied using micromechanical test, HR-EBSD and HR-DIC characterization, and crystal plasticity FEM. Explicit representation of the experimentally characterized microstructure of the agglomerate system has been developed. Crystal plasticity FEMs were used to predict local stress and strain conditions within the microstructure, and particularly local to the agglomerate inclusion. The residual (elastic) strains and dislocation distributions resulting from the initial heat treatment of the sample have been found to have negligible effect on subsequent low cycle fatigue. The CPFE predicted distributions of plastic strains within the microstructure are in good agreement with the HR-DIC measurements both qualitatively and quantitatively.

A range of micromechanical quantities (hydrostatic stress, effective plastic strain and stored energy) are available from this microstructure-sensitive model. Direct comparisons of model results with experimental observations demonstrate that nickel/agglomerate interface decohesion is directly and unambiguously related to the normal tensile stress across the interface. Interfacial strength extracted from the coupled crystal plasticity—micromechanical test was found to be in the range 1270–1480 MPa.

## Figures and Tables

**Figure 15.**
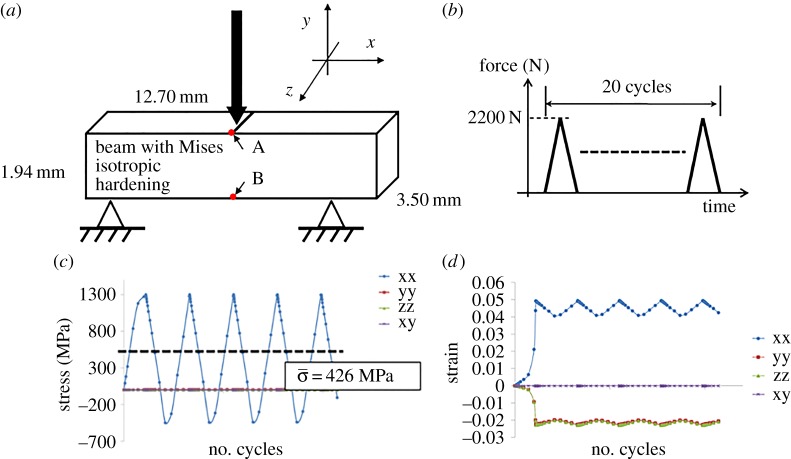
Schematic of the three-point bending beam modelled using Mises isotropic hardening plasticity, (*b*) the applied fatigue loading of 20 cycles with peak force of 2200 N, (*c*) stress and (*d*) strain response at the point B during the first five loading cycles. (Online version in colour.)

**Figure 16.**
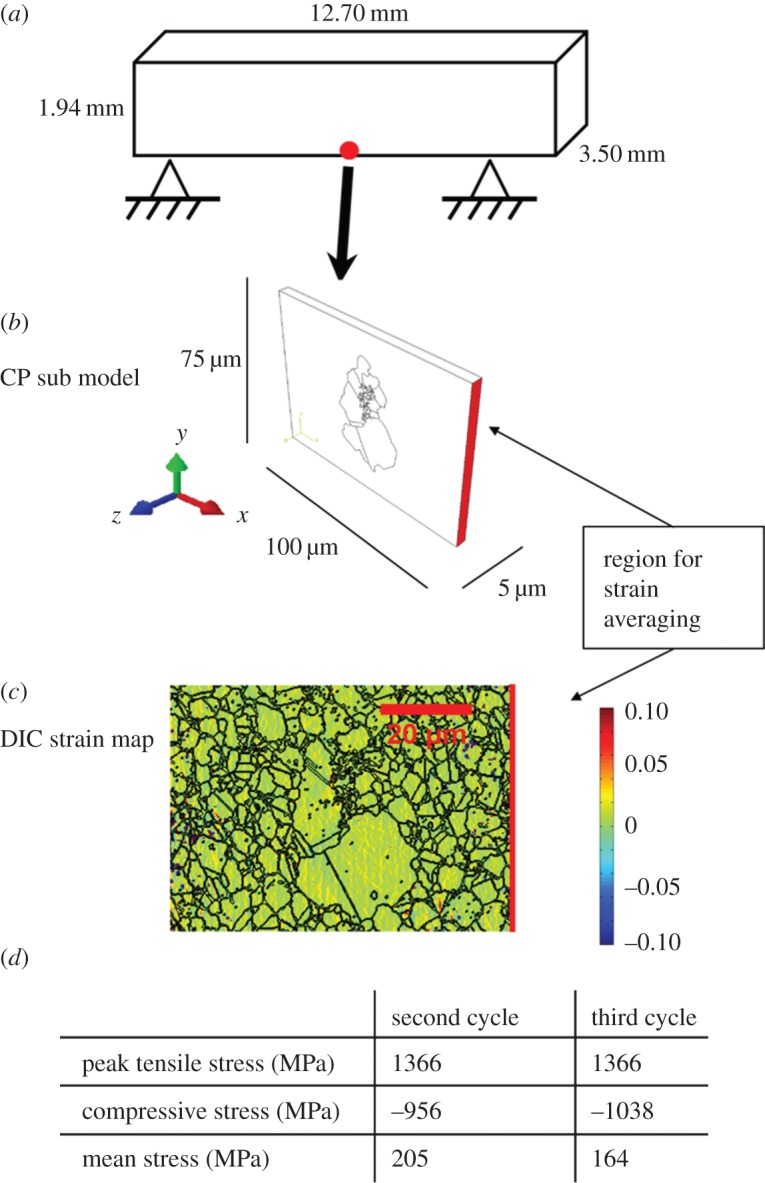
(*a*) Schematic of the macrolevel three-point bending beam. Regions over which xx strains were averaged in (*b*) the crystal plasticity submodel of the macro model and (*c*) DIC strain map at the unloaded state of the end of cycle 20. (*d*) The magnitudes of the final peak tensile, compressive and mean stresses applied (to give the result in figure 2*f* for mean stress A). (Online version in colour.)

## Data accessibility

The experimental results reported in this paper can be found online at https://zenodo.org/deposit/69345/.

## Appendix A

It is computationally challenging to model the entirety of the three-point beam experiment using crystal plasticity with full representation of the details of the agglomerate and surrounding microstructure. As a result, a submodelling methodology is developed in which the crystal representation of the microstructure is faithfully implemented for a small region of the three-point beam test and the boundary condition of this submodel is determined using a large-scale Mises plasticity model. This is the subject of this appendix, which firstly outlines the Mises plasticity full-beam modelling, and second, the parallel submodel crystal plasticity representation in order to establish the correct local boundary conditions.

**(a) Mises plasticity modelling of the three-point beam bending**

An FEM with isotropic hardening J_2_ plasticity was developed for the macrolevel beam which, with the applied boundary and loading conditions, is shown schematically in figure 15*a* where 20 cycles of force-controlled cyclic loading in figure 15*b* was applied to reproduce the loading history in the experiment.

The stresses and strains at point B predicted by the Mises FE model are plotted in figure 15*c* and *d*, respectively. The xx stress first increases linearly and then becomes plastic, followed by an elastic unloading. Analysis of the collective results demonstrates that point B is deformed effectively under uniaxial loading and that the effects of shear are negligibly small (as anticipated near the beam free surface). In addition, the area of the region of interest associated with the agglomerate inclusion at point B is small enough such that over the region where local boundary conditions are to be imposed on the submodel, the applied stress loading is uniform as well as uniaxial.

Because the model employs Mises isotropic hardening, a load reversal does not cause translation of the yield surface and thus over-predicts the elastic region during this load reversal. As a result, the isotropic hardening model generates constant positive mean stress as indicated in figure 15*c* and no strain ratcheting is predicted. However, HR-DIC strain measurement carried out in [20] has shown that the cyclic loading does indeed cause significant strain ratcheting at the microstructural level. This is because there exists a non-zero mean stress which, during cyclic loading, drives incremental plastic ratcheting. At point B in the beam, the mean stress is unknown because of the stress relaxation which occurs over the first few cycles, though is bounded by the known tensile stress at peak loading and zero (which would reflect the applied *R*=0 loading). Hence, in order to determine the appropriate minimum stress, and hence mean stress, to apply, the experimentally (HR-DIC) measured total strains at point B are used. A crystal plasticity submodel, fully representative of the agglomerate microstructure at point B, is subjected to cyclic stress-controlled loading with peak stress defined above. The minimum stress, however, is chosen in the submodel to lead to the correct level of plastic ratcheting measured at point B by DIC in the experiment. The submodel is described next.

**(b) Crystal plasticity submodelling**

In figure 16*a*, a subregion of the three-point bending beam containing the inclusion agglomerate was modelled using crystal plasticity. The submodel with its dimensions is shown in figure 16*b*. The submodel is sufficiently small such that the applied stress variation from the beam bending across the region can be neglected. That is, uniform, uniaxial cyclic applied stress is appropriate. The boundary conditions applied to the submodel are such that the left face is fixed in the *x*-direction, the back face is fixed in the *z*-direction, and the bottom face is fixed in the *y*-direction. Cyclic stress loading is applied on the right face.

In order to obtain the correct loading conditions for the submodel as discussed above, uniaxial stress loading with fixed peak tensile stress, but various different mean stresses were applied on the right face (coloured in red in figure 16*a*) in the *xx*-direction. Example resulting average strains from the submodel are plotted in figure 2*f*, labelled mean stress A, together with averaged DIC-measured xx strains over twenty cycles of stress-controlled loading. Averaging of xx strains (at unloaded states of the second and twentieth cycles) was carried out in the DIC and crystal plasticity submodel as shown in figure 16*b*,*c*. In the crystal plasticity submodel, nodal x-displacements on the right face were averaged and converted into corresponding strains. In the DIC, maps of xx strains at unloaded states of the second and twentieth cycle were used for strain averaging as shown in figure 16*c*. The strain map is identical to the crystal plasticity submodel in size. Average strain was calculated over the region on the far right of the map. Because the inclusion agglomerate has a relatively small size (approx. 8 μm) compared with that of the map (100 μm), its contribution on the average strain is negligibly small and hence the average strain calculated is representative.

The mean stress chosen to be applied to the submodel was that which gives closest agreement with the experimentally DIC-measured ratchet strains, marked by the crosses in figure 2*f*. The chosen boundary conditions correspond to the calculated strain evolution in figure 2*f* labelled mean stress A.
